# Business for ocean sustainability: Early responses of ocean governance in the private sector

**DOI:** 10.1007/s13280-022-01784-2

**Published:** 2022-10-19

**Authors:** Rafael Sardá, Stefano Pogutz, Manlio de Silvio, Virginia Allevi, Aristea Saputo, Roberta Daminelli, Federico Fumagalli, Leonardo Totaro, Giorgia Rizzi, Giulio Magni, Jan Pachner, Francesco Perrini

**Affiliations:** 1 Centre d’Estudis Avançats de Blanes (CEAB-CSIC), Carrer d’acces a la Cala Sant Francesc, 14, 17300 Blanes-Girona, Spain; 2 Bocconi University, Via Roberto Sarfatti, 10, 20136 Milan, Italy; 3 McKinsey Global Institute, McKinsey & Company, Piazza del Duomo, 31, 20122 Milan, Italy; 4 One Ocean Foundation, Via Gesù 10, 20121 Milan, Italy

**Keywords:** Blue economy, Corporate sustainability, DPSWR analysis, Ocean disclosure practices, Ocean sustainability

## Abstract

A large sample of 1664 companies—69 directly working in the ocean economy—distributed across 19 industrial sectors was investigated to explore awareness and activation regarding direct and indirect pressures on the ocean, their responses to these pressures, and the disclosure tools used. We examined their accountability and disclosure practices on sustainable development goals (SDGs) using the drivers, pressures, state, welfare, and response accounting framework. Based on their 2019 sustainability reports, just 7% of the companies assessed disclosed on SDG14. However, 51% of these companies can be considered as aware, albeit to varying degrees, of the pressures their industries place on the oceans, 44% deploy mitigating activities, and 26% are aware and actively lead business responses to ocean challenges. Although we have seen just early responses in addressing ocean challenges, companies’ awareness and activation must converge to achieve ocean sustainability and move businesses into a truly blue economy.

**Supplementary Information:**

The online version contains supplementary material available at 10.1007/s13280-022-01784-2.

## Introduction

Our human footprint is threatening the health of the oceans and seas—and major threats include: ocean warming and acidification (IPCC 2019); overfishing and bycatch (FAO 2020); pollution and litter (Barnes et al. 2009); and eutrophication and anoxic episodes (Breitburg et al. 2018). Mirroring terrestrial systems, large declines in species population (McCauley et al. 2015; IPBES 2019; IUCN 2022) and habitat loss (Langmead et al. 2007) are indications of overall marine ecosystem health, and these events are sending us flashing red warnings about the state of the seas and oceans (see last revision in UN 2021a, b). The oceans are our planet’s largest life-support system and, the future health of human populations depends on their healthy state (Borja et al. 2020); however, the demand for ocean resources will grow, and as land-based sources decline, so expectations for the ocean as an engine of human development for food, materials, and space will increase (Jouffray et al. 2020; Virdin et al. 2021). Preserving the health of marine and coastal ecosystems is paramount because of the many irreplaceable benefits they provide.

When holistically considering the oceans as a social-ecological system we see that their health depends on (Rapport 1998): (a) the biophysical dimension, the basic properties of an ecosystem, its structure and functions (nutrient cycling, energy flows, sequestering of toxic substances or habitats, and biodiversity) that provide ecosystem goods and services on which human societies depend; (b) the human health dimension incorporating our exposures to natural risks, but also our dependence on socio-economic activities and the need to balance economic viability with conservation, and finally; (c) the spatial and temporal dimension that encompasses life cycle analysis and long-term thinking, and which is basic for human reliance on sustainable development. The 2021s World Ocean Assessment (UN 2021a, b) warns us that the greatest threat to the ocean is the failure to deal with the many pressures caused by human activities, and advises us that to ensure sustainability, we must work together to improve integrated ocean management, including through joint research, capacity development and the sharing of data information and technology. However, tracking the global results of an Ocean Health Index for exclusive national economic zones, results indicate a trend of slow improvement (Halpern et al. 2015; OHI 2021). A recent paper (Duarte et al. 2020) also strongly supports the idea that by rebuilding marine life, a substantial recovery in the abundance, structure, and function of marine life can be achieved by 2050 if major pressures including climate change are mitigated. The challenge is how to diminish these pressures.

The ocean economy however, is rapidly growing as commercial use of the ocean accelerates (Jouffray et al. 2020; Virdin et al. 2021). If oceans are just seen as an engine for future economic growth following “business as usual scenarios” it is clear that this vision collides with the present vision for the governance of the oceans and its global ocean conservation policies following the United Nations Convention on the Law of the Sea, the Agenda 21 of the 1992 United Nations Conference on Environment and Development, the United Nations 2030 Agenda for Sustainable Development, or the recent works searching for a Treaty of the High Seas. In this context, the private sector is increasingly recognized as having the capacity to hamper efforts to achieve aspirations of sustainable ocean-based development or alternatively to bend current trajectories of ocean use by taking on the mantle of corporate biosphere stewardship (Virdin et al. 2021).

By following science-based principles (Costanza et al. 1998), we adopt the concept of ocean sustainability as the approach required to manage our oceans and the services they provide. Ocean health was long self-regulated and maintained, but man-made pressures today pose a threat (UN 2021a, b). We need to diminish these pressures to rebuild marine life and provide the conditions for a resilient and functional ocean (Duarte et al. 2020). Improving the international ocean governance framework is needed, however, private companies delayed addressing ocean challenges for decades and now, they must reduce their pressures on the oceans in an accelerated way if we want to advance altogether to the desired blue economy. As a novelty, this research addresses the early responses in the private sector to reduce human pressures on the oceans and to create the conditions for a blue economy, an aspirational goal in the coming decades to address ocean challenges. The Blue Economy (UNCSD 2012) was thought to initiate a transformative process that shall allow traditional marine activities (“Ocean-Based Economy”) to be carried out in the future without compromising the proper functioning of the oceans and its provision of ecosystem goods and services, while promoting at the same time equity and social welfare. However, there is a lot of ambiguity in the way the Blue Economy term is used today; in this paper we take a clear position on what the Blue Economy is and what it is not, distinguishing the traditional ocean economy (“Ocean-Based Economy”) from the Blue Economy (“Blue Economy”). Following this position, the term "Sustainable Ocean-Based Economy" would be synonymous with the "Blue Economy" and the term "Sustainable Blue Economy" should not be used.

The main objective of this paper is to investigate the early responses of the private sector to these pressuring trends. We assess corporate awareness and activation strategies with the focus on the ocean issues. We investigate the direct pressures generated by industrial and consumption activities, as well as the indirect pressures on marine and coastal ecosystems, thus extending the boundaries of the analysis. We acknowledge that ocean sustainability will emerge when both terrestrial and marine-based activities operate in balance with the long-term capacity of their ecosystems to support them while remaining resilient and healthy.

## Methodology

In the oceans, a challenge exists between conservation and sustainable use. Since 2015, a healthy and productive ocean has been the principal consideration addressed in the Sustainable Development Goal 14 (SDG-14 ‘Life Below Water’) in the context of the United Nations 2030 Agenda for Sustainable Development (UN 2015). The main goal of SDG14 is “*to conserve and sustainably use the world’s oceans, seas and marine resources for sustainable development*”. In this paper, the vast planetary ocean system is considered as a large social-ecological system [see Supplementary Material 1 for a detailed description].

After valuing the present and traditional ocean economy, a science-based analysis of direct (pressures occurred through a direct interaction of an activity with an environmental component in the sea) and indirect (pressures coming for this interaction but occurring off the sea) industrial pressure on ocean health is presented. First, to assess the relationship between human pressures and the state of the marine environmental component, we will use the European Union (EU) Good Environmental Status (GEnS) as a conceptual framework, “*the environmental status of marine waters where these provide ecologically diverse and dynamic oceans and seas which are intrinsically clean, healthy and productive, and the use of the marine environment is at a level that is sustainable, thus safeguarding the potential for uses and activities by current and future generation*s’’ (European Commission-EU 2008; Borja et al. 2010). Secondly, we assess business commitment to the different United Nations Sustainable Development Goals-SDGs (UN 2015) comparing SDG 14 (‘Life Below Water’) to other SDGs as a snapshot of the level of attention among ocean economy companies to this specific SDG. Finally, an analysis of ocean-related awareness and activation strategies and the disclosure practices of global companies was made. Altogether, our final goal was to explore business activities to better capture the level of attention given towards marine issues, and to assess the development of coherent responses by corporate sustainability leaders.

Understanding this social-ecological system as the interaction of two co-evolving sub-systems should enable us to find the right responses to the environmental challenges we are facing. A general framework for the analysis of social-ecological system sustainability was proposed by Ostrom (2007, 2009). The framework is intended to organise the entire system (applied in our case to the ocean), its resources, users, and governance rules. Scientists (McGinnis and Ostrom 2014) have emphasised points in its nested structure (physical parts of the system and its relations). Our hypothesis here is that without the implication and collaborative action of private companies (a particular type of user in the system) to deal with the sustainable management of its natural resources, it is going to be difficult to tackle the challenge and contribute to finding solutions to address degradation and resource depletion.

The methodological logic behind this paper follows the social-ecological accounting framework driver-pressure-state-welfare-response (DPSWR) (Cooper 2013), a modification of the driver-pressure-state-impact-response (DPSIR) (EEA 1999), which we believe is relevant when connecting social and natural sub-systems to assess the benefits of nature obtained by societies. Recently, this framework has been updated in a newer version (Elliott et al. 2017) that facilitates more detailed analysis. The word ‘impact (I)’ is used for policy and practice both to assess corporate footprints and dependence on environmental issues (making this word a confusing element when different people coming from different disciplines discuss it together). The world ‘welfare (W)’ captures the benefits that nature gives man and defines better the narrative to be employed. According to the DPSWR approach, social sub-systems (industrial sectors and their activities), are  <drivers>  of change (D). They put <pressure> (P) on natural sub-systems, structural units, and their functions in nature that can alter their <state> (S) because of these pressures. This process, in turn, can translate into the degradation of fundamental natural resources used by humans (natural goods and ecosystem services), thus diminishing the benefits for human  <welfare> (W). The acknowledgment of such a process of degradation should induce humans to develop adequate   <response> (R), for example, policies and innovative solutions that address the ecological problems, reduce pressures, and help restore social-ecological system resilience [see Supplementary Material 1]. These two sub-systems are interconnected basically in two ways. On one side, social sub-systems pressure natural sub-systems (generally in a negative way), and this is the main reason for the challenging aspects. On the other side, we are largely dependent on a complete array of ecosystem goods and services coming from the natural sub-system. When we analyse these interconnections, they will only be ‘acceptable’ if natural resources are used at the rate that they can regenerate themselves to maintain through time the provision of ecosystem goods and services in a resilient manner (Sardá and Pogutz 2019), and this constitutes the basic ocean global environmental challenge for the future.

In this paper, we will not assess the   <state> (S) element other than the EU Good Environmental State (Borja et al. 2010) framework used for the analysis. The state of ocean and seas have been largely assessed in multiple papers and reports (last review in UN 2021a, b). Instead, we will concentrate the analysis on the footprint aspect  <driver-pressure> elements, and overall, on the dependence aspect < welfare-response> elements. The analysis is based on data analysed through quantitative and qualitative research methods that are explained below.

### <Driver-pressure> assessment

The value of the global ocean economy, measured in terms of the contribution of the ocean-based industries to the economic output (annual revenues, and Gross Value-Added-GVA) and employment, was computed by carrying out initially, an extensive review. This was done by gathering and comparing information from many sources among both academic papers an grey literature (World Bank 2013; OECD 2014, 2016; FAO 2020; Eurostat 2020; WTTC 2020; Rystad 2021; HIS Markit 2021).

For the < driver> element, a large sample of 1664 companies distributed across 19 industrial sectors and accounting for about 50% of the world’s market capitalisation was analysed. A total of 69 of these companies operate directly in the ocean economy. The ocean economy sectors, in line with the definitions adopted by the OECD (2016) and the World Bank (2017), encompass well-established sectorial economic activities (i.e. coastal tourism, commercial fishing, industrial aquaculture, shipbuilding and ship maintenance, offshore oil and gas extraction, port activities, shipping, and maritime transport) and other emerging sectors (i.e. exploitation of marine renewable energy, the use of marine biodiversity for medical pharmaceutical purposes, desalination, and seabed mining). For this paper, ocean economy sectors have been bundled into three groups: extractive renewable (fisheries and aquaculture); extractive non-renewable (seabed mining, offshore oil, and gas); and operational (transportation, ports and warehousing, shipbuilding and repair, coastal tourism, desalination, renewable energy, genetic and medical resources) [See description of all these groups in Supplementary Material 2a]. To reduce the pressures on marine ecosystems, significant changes in all the above sectors are needed today.

To target the response of the European Union (EU) to the present health of the oceans and seas, the EU established the goal of achieving GEnS for its marine environment, a goal introduced and defined by the EU Marine Strategy Framework Directive (2008/56/EC) (Borja et al. 2010, 2013). The GEnS concept is defined through various indicators related to 11 descriptors [see a description in Supplementary Material 2b]. The basic idea is that the cumulative pressure of all human activities affecting the European seas (direct and indirect pressures) should not impede reaching the indicator targets selected for GEnS. As pointed out in the Commission Decision 2017/848/EU (European Commission 2017), the criteria for the achievement of GEnS is the starting point for the development of coherent approaches in the preparatory stages of marine strategies, including the determination of the characteristics of GEnS and the establishment of a comprehensive set of environmental targets to be developed in a coherent and coordinated manner in the framework of regional cooperation. All planned activities in the EU marine domain should cumulatively not put GEnS at risk. Although GEnS have only been introduced as a mandatory requirement within the boundaries of the EU, this approach could be applied to all world coastal states, and we used its vision for this research as a philosophy to be considered when guiding a desired vision for the future of world marine waters.

The < pressure> element was analysed based on its footprint in the 11 GEnS descriptors. Direct and indirect pressures from production and consumption activities, both ocean and non-ocean related, have consequences on marine ecosystems. In addition, direct and indirect pressures include cumulative effects, since the pressures on environmental resources may result from changes determined by past, present, and future actions, as well as from their interactions [in the Supplementary Material 3 we reviewed all these pressures exerted on the 11 descriptors of GEnS]. In this paper, we consider a company-based pressure on the environment ‘*A means by which the company at least causes or contributes to a change in state*’ (Cooper 2012). Pressures can occur through a direct interaction of an activity with an environmental component (e.g. sea-floor integrity endangered by oil and gas drilling, sea-bed mining, trawling, etc.). Indirect pressures can be observed through an indirect interaction of an activity with an environmental component (e.g. Greenhouse Gas emissions –GHG- determining an increase in sea temperature and acidification). In both ways, these pressures can be observed in space at a micro-level (local area of impact—such as a site, a bay, and so on); meso-level (regional area of impact—such as a region or a basin); or macro-level (global area of impact—such as an ocean and atmosphere).

Determining and managing the effects of human activities (changes in the states of nature because of human-induced pressures) requires a risk assessment and management approach in which decision-making often occurs in the absence of information, or the presence of poor information that increasingly requires expert judgment (Elliott et al. 2013). A panel of 56 natural and social scientists with different natural science backgrounds and from leading research institutes and universities across Europe, North and South America, and Australia was involved [the composition of the panel can be seen in Supplementary Material 4a and the questions from the survey in Supplementary Material 4b]. The purpose of the survey was to collect ocean expert opinions about the relevance of direct and indirect pressures exerted by 17 of the 19 industrial sectors assessed on the GEnS descriptors. We asked the experts to unfold their perceptions or opinions across these industrial sectors of interest (in this case we added to the ocean economy sectors, other land-based indirect sectors pressuring oceans and seas), through a Likert scale score (1 to 7). To simplify the given information in this paper, we translated the average quantitative data of the Likert scale into four possible qualitative pressures: (a) no pressure—light blue (1–2.5); (b) low pressure—light yellow (2.5–4); (c) medium pressure—orange (4–5.5); and (d) high pressure—red (5.5–7).

### <Welfare-response> assessment

Ocean and coastal ecosystems provide people, societies, and businesses with a wide range of essential goods and services (i.e., coral reefs attract tourists, serve as nurseries for commercial fish species, and protect coastline properties from storm surges). At the base of this relationship lies the concept of ecosystem services: ‘*the benefits man obtains from nature*’ (MEA 2005; Hanson et al. 2012). Services provided by the ocean make a major contribution to our economic and social development and include food and freshwater supply, renewable energy, benefits for health and wellbeing, cultural value, tourism, trade, and transport. Businesses now are starting to acknowledge this contribution and to consider that these benefits (our < welfare> element) could have limits if resilience is not ensured.

To investigate business responses to the challenges of ocean sustainability we analysed the sustainability reports published in 2019 in a sample of 1664 companies. Sustainability reporting have become widespread among large multinational companies. A recent publication by KPMG (2021) shows that 80% of the N100 companies, where N100 refers to the top 100 companies (large and mid-cap firms) in 52 different countries around the world, publish a sustainability report. The same survey shows that 96% of the top 250 companies by revenues among the Fortune 500 rankings publish non-financial reporting. This trend can be seen as the result of pressures for disclosure and transparency on corporate sustainability practices by multiple stakeholders and regulatory bodies [e.g. the EU Directive 2014/95 on non-financial disclosure, the proposal for a Corporate Sustainability Reporting Directive and the Environmental, Social and Governance (ESG) disclosures introduced by the Sustainable Stock Exchanges}. The growing attention to sustainability reporting can be also linked to the development of global standard settings such as Global Reporting Initiative (GRI), the International Integrated Reporting Council (IIRC), the Sustainability Accounting Standards Board (SASB), and more recently by European Financial Reporting Advisory Group (EFRAG) and the International Financial Reporting Standards (IFRS) (Afolabi et al. 2022). In accordance with these frameworks and standards, in our analysis we adopted a broad approach to exhaustively cover the different types of non-financial information published by companies (Hahn and Kühnen 2013; Amini et al. 2018).

In the last decades, also in the academia sustainability reporting has become an increasingly relevant topic for research in disciplinary domains such as management, accounting and finance, business ethics and sustainability (Elkington 1997; Kolk 2010; Lozano and Huisingh 2011; Hahn and Kühnen 2013; Amini et al. 2018). This literature has investigated why and how companies disclose ESG information finding links with reputation and legitimacy, investors’ expectations, financial risks and corporate value, motivation of employees, increased control over processes and information flows, support to management decisions. Moreover, a broad research stream has focused on investigating sustainability reporting as an important tool to contribute to corporate sustainability (Burritt and Schaltegger 2010; Lozano and Huisingh 2011).

These studies have also offered a critical perspective on sustainability reporting, showing that this practice still requires more standardization, increased transparency, and quality improvements (Dando and Swift 2003). Anyway, the introduction of quality assurance practices (Gürtürk and Hahn 2016; Boiral et al. 2019) and the diffusion of standardized guidelines (e.g. GRI, SASB, IIRC) and mandatory frameworks (EU Directive 2014/95) have become more diffused, consolidating the utilization of non-financial reporting as a methodology to screen and investigate corporate sustainability awareness and behaviour for the exam of ESG practices in different industries. For example, Weber and Marley (2012) used sustainability reporting to analyse stakeholder salience and corporate social responsibility practices. Amini et al. (2018) used content analysis to investigate non-financial reports and map corporate sustainability definitions and frameworks. More recently, Opferkuch et al (2020) analysed circular economy approaches and business models by a sample of international companies, building on the data and information contained in their sustainability reporting.

Following this approach, to do the analyses we used databases provided by the Datamaran company (https://www.datamaran.com/), the market leader in external risk management and in capturing ESG reporting activities. The sample was made up of companies belonging to 19 industrial sectors, including 69 organisations from ocean economy sectors as described previously. In terms of economic contribution, the sample represents companies with a total market capitalisation of almost $45 trillion.

The study was carried out with natural language processing and lexicometric analysis. Natural language processing (NLP) is a relatively new but well-established methodology that combines content analysis methodology with the power of artificial intelligence, computational linguistics, and computer science, to help understand and sort various words (variables) in texts [the words used for each of the variables assessed in this work can be found in the Supplementary Materials 6]. With the advent of big data, data-driven NLP allowed to effectively manage the complexity of analysing large amount of text using large datasets to build high-quality models (Gudivada and Arbabifard 2018).

We analysed how many in each sample (1666 in total, 69 in ocean economy sectors) mentioned the word ‘ocean’ (variable 1) and analysed how companies disclose information on SDGs, the “SDG 14” (variable 2), and made a comparison with the rest of the mentioned SDGs.

To perform an analysis of awareness (variable 3) and activation (variable 4), a second sample of 626 companies—those out of the 1664 that mentioned the word ‘ocean’ or ‘marine ecosystems’ or other combinations in their reports—was used. In this case, the selected firms belonged to 13 sectors and represented a total market capitalisation of $15 trillion (more than 17% of global market capitalisation).

In this case, a combination of more than 200 keywords, reflecting corporate awareness and activation with regard to the marine environment, were selected through several rounds of expert consultation [see Supplementary Material 4a]. In particular, the final set of keywords was selected through a three-step process, developed to operationalize the variables, and ensure that items were grounded in the theory and relevant for the purpose of the analysis. In the first step, an initial set of keywords was identified through a thorough literature review. In the second step, keywords were independently validated by researchers and practitioners not involved in the first phase. In the third step, the final sample of keywords was reviewed for consistency check and validation.

The operationalization of the ‘awareness’ variable was achieved through the selection of keywords related to the acknowledgment of ocean-related issues, such as, for example, retrieving in the analysed sustainability reports the keyword ‘acidification’ in proximity (i.e. within ten lexical items) of ‘ocean’ or ‘marine’ or other combinations of lexical constructs (e.g. ‘coastal’ or ‘seawater’). The operationalization of the ‘activation’ variable was achieved including in the query additional keywords expressing the implementation of actions to prevent or mitigate the pressures exerted by companies, or to restore negative effects (e.g. ‘reducing’ or ‘avoiding’ or ‘preventing’ and similar lexical constructs). Variables were assigned positive binary values in case of presence of combination of keywords related to the acknowledgment of pressures on the ocean, or in case of presence of combination of keywords inherent to the activation in favour of marine preservation, mitigation of pressures, restoration of negative effects.

Advanced lexicometric methodologies and ad-hoc scoring systems were used to extract and prepare the data to map and analyse the extent of corporate awareness and the initiatives adopted to mitigate their pressures on marine ecosystems and match these variables with the opinion of the experts.

We define companies as being ‘aware’ of the negative pressures directly and indirectly exerted by their activities on marine and coastal ecosystems when their acknowledgment (i.e. when the variable was assigned with a positive binary value) matches the opinion of ocean science experts (i.e. when the expert opinion of the severity of the pressures reaches at least the medium of high value).

We define companies as being ‘active’ when the variable is assigned with a positive binary value indicating that product development, process innovations, and supply chain solutions, even when indirectly related to ocean health, are adopted, and thus can help companies mitigate their pressures on marine ecosystems.

Finally, we conclude the analysis by assessing the distribution of the most used transparency and disclosure standards (variable 5), initiatives, and frameworks in non-financial reports. To map the orientation towards general, as well as more issue-specific forms of transparency and disclosure, we tested how frequently companies mentioned a broad range of standards, initiatives, and frameworks.

## Results

### <Driver>: the value of the ocean economy

The global ocean economy, measured in terms of the contribution of the sectors of the ocean economy to economic output and employment is significant. According to our calculations, the value of the global ocean economy in 2017 (the year for which we have a complete set of comparable data) was around $2.6 trillion calculated in Gross Value-Added (GVA), or approximately 3.3% of the world gross domestic product (GDP), making the ocean the world’s seventh-largest economy. It generated estimated annual revenues of $5.2 trillion and employment for 168 million people.

**Fig. 1:**
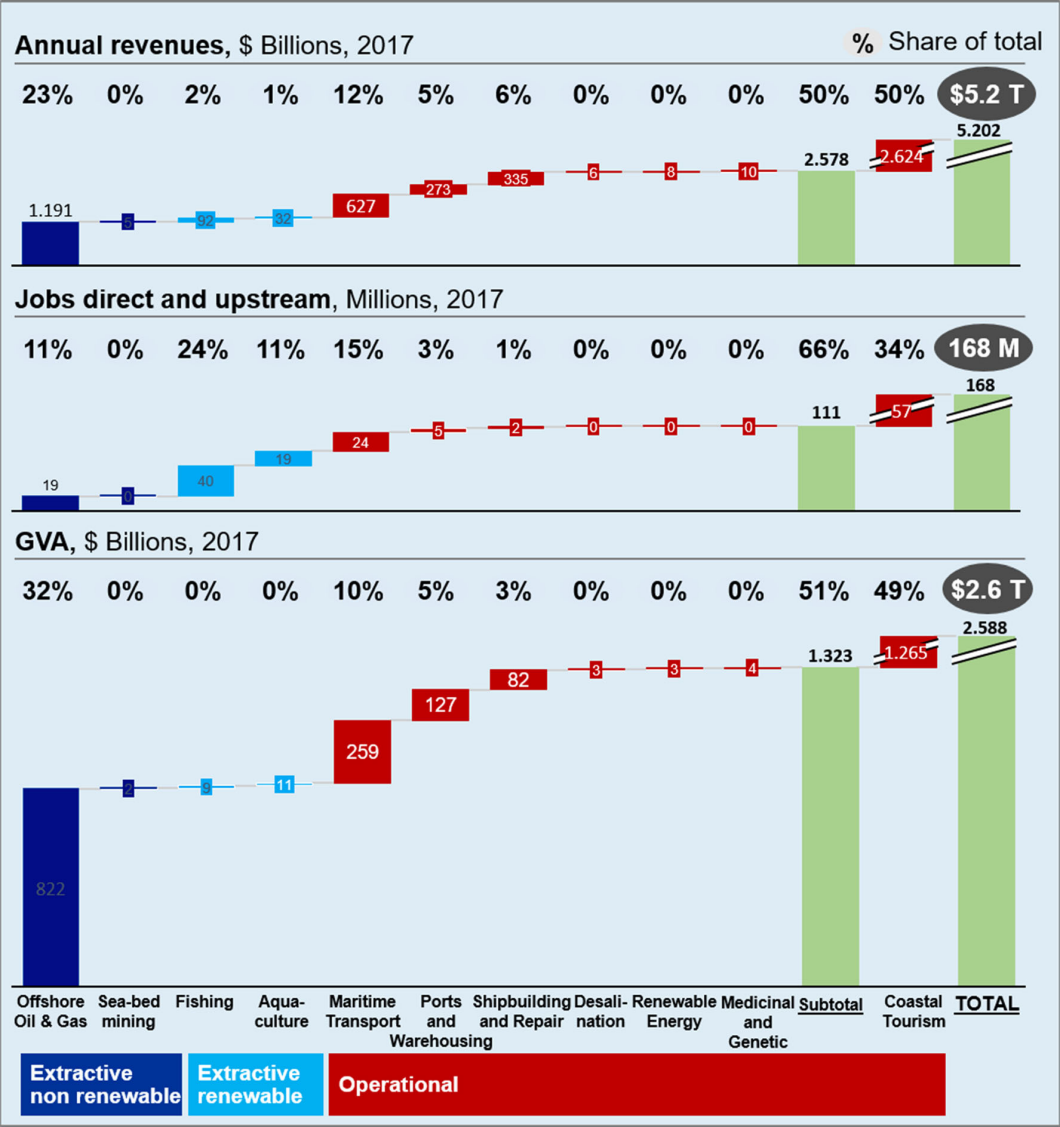
Ocean economy data by industrial sectors (2017 data)

Among the established ocean economy sectors, coastal tourism (the most relevant activity both in terms of annual revenues and employment) accounted for half of the total ocean economy value-added, followed by offshore oil and gas (32%), maritime transport (10%), port and warehousing activities (5%), and shipbuilding and repair activities (3%) (Fig. 1). Ocean economy industries provided 168 million jobs, with the largest employers being coastal tourism (34%), fisheries (24%), aquaculture (12%), and maritime transport (15%). The economic value of emerging and innovative sectors (i.e. marine renewable energy, desalination, seabed mining, and genetic and medical resources) was still limited at around just 0.5% of the total but their potential was considered to be high.

Each of the world’s oceans (the Atlantic, Arctic, Indian, Pacific, and Southern Oceans) as defined by the International Hydrographic Organization (IHO) has its own specificities. Therefore, we conducted a regional analysis to provide an overview of the key socioeconomic features of each ocean. A detailed regional analysis of the three ocean economy groups based on the GVA from their activities (2017 data) is shown in Table 1 (stated for the groups of sectors involved in the analysis). In 2017, the Atlantic and Pacific oceans generated almost $1.8 trillion of GVA; this represents 70% of the overall global ocean economy value added [see Supplementary Material 7 for a graph]. In terms of employment, the Pacific Ocean has the largest share with 82.2 million employees (49% of the world ocean total), followed by the Indian (27.2%), and Atlantic (20.9%) oceans.

**Table 1:** Ocean economy groups based on the gross value added (GVA) assigned to world’s oceans (2017 data)

Ocean	GVA ($ bn) [jobs (Millions)]	Industrial sector groups	GVA ($ bn)
Arctic Ocean	195 [4.5]	Extractive non-renewable	28.5
		Extractive renewable	–
		Operational	168.4
Atlantic Ocean	945 [35.1]	Extractive non-renewable	248.6
		Extractive renewable	2.6
		Operational	691.5
Pacific Ocean	890 [82.2]	Extractive non-renewable	189.1
		Extractive renewable	15.5
		Operational	681.2
Indian Ocean	555 [45.7]	Extractive non-renewable	354.8
		Extractive renewable	2.6
		Operational	196.8
Southern Ocean	10 [0.3]	Extractive non-renewable	5.2
		Extractive renewable	–
		Operational	5.2

### <Pressure>: perceptions from science

**Fig. 2:**
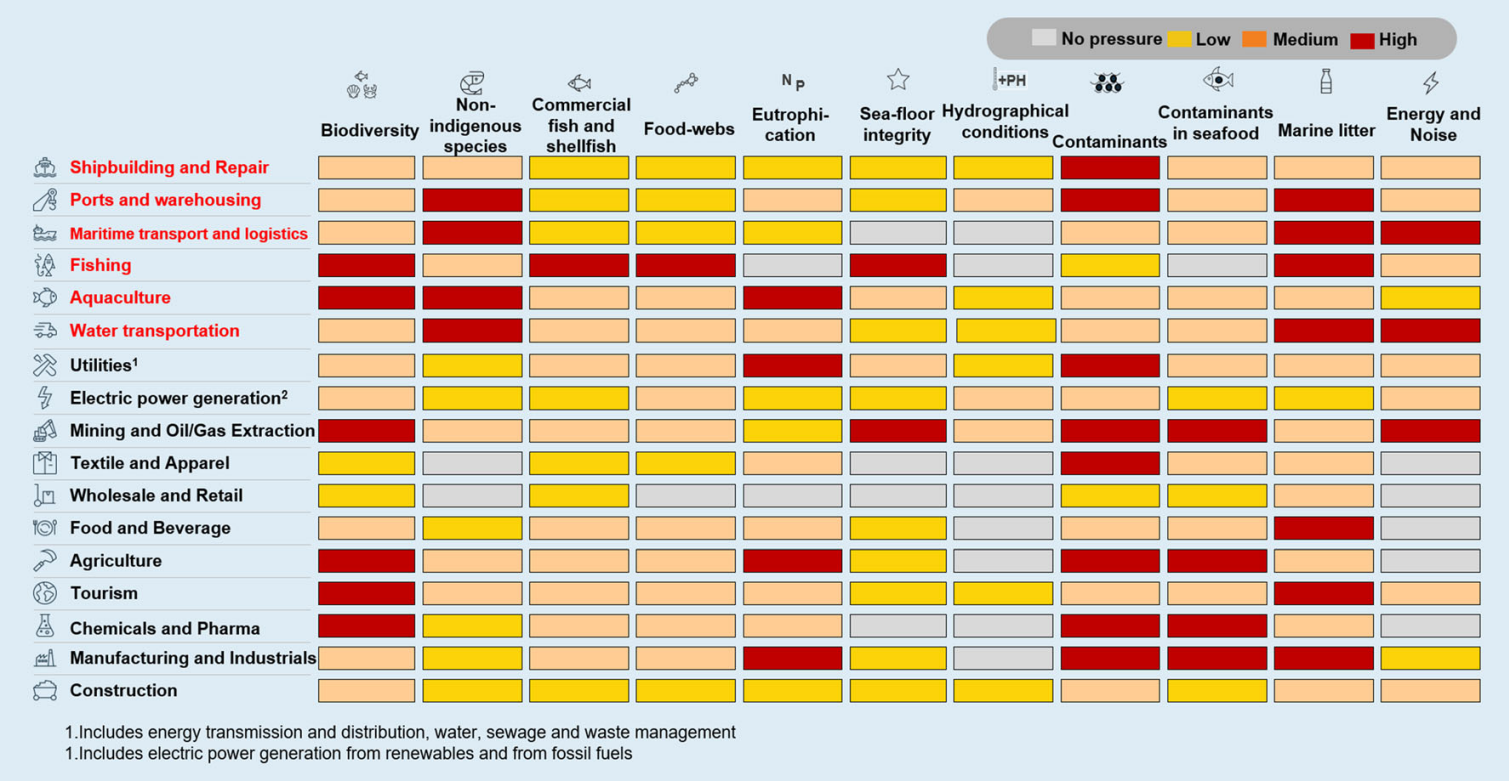
Expert judgment analysis of the pressures imposed by 17 industrial sectors on good environmental status (GEnS) (in red industrial sectors operating on the ocean economy)

The questionnaire completed out by the ocean experts revealed that all industries directly or indirectly interacting with the ocean/seas potentially exercise negative pressures on most of the 11 GEnS descriptors (Fig. 2). From this perspective, the scientific review confirmed the most significant pressures for ocean health as being those related to:

- Effects on marine biodiversity, including depletion of fish stocks and alteration of food webs, also co-determined by several different causes, such as the modification of the hydrographical condition of waters, pollution, eutrophication, and the alteration of seafloor integrity.
- Introduction of contaminants in marine ecosystems, including their presence in seafood, either through direct interaction with the marine environment, or indirectly through wastewaters, discharge points, or atmospheric deposition.
- Pollution of the ocean and marine environments through the discharge of litter and other human-created waste, being plastic the main worry (GESAMP 2015); as extensively reported by several different studies (Ocean Conservancy and McKinsey 2015).

### <Welfare>: reporting on SDG14 dependences

**Fig. 3:**
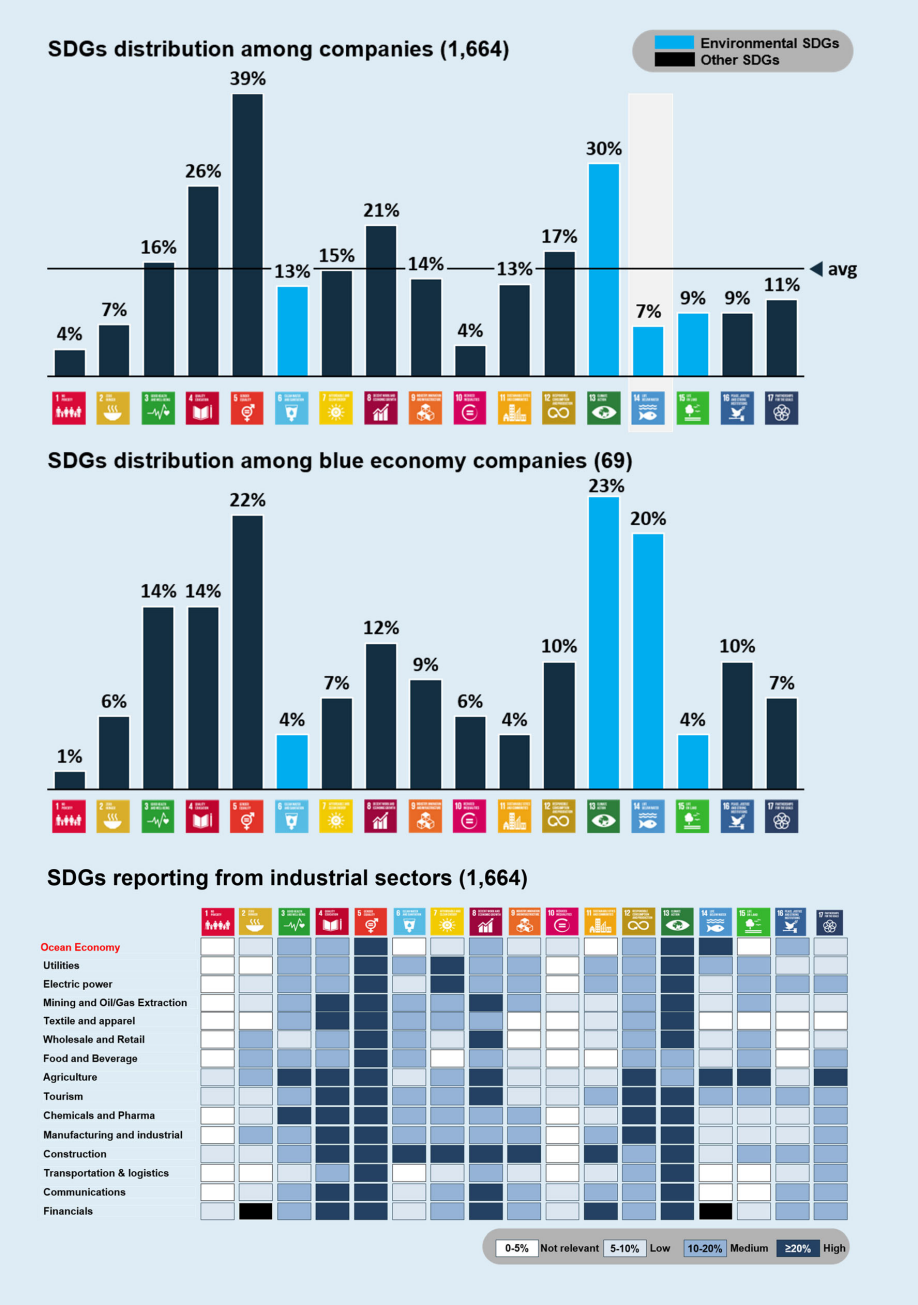
Percentage of mentions of the different Sustainable Development Goals (SDGs) in their 2019 sustainability reports. Upper graph, a global analysis with the total sample (1664 companies). Middle graph, same analysis only with the ocean economy sectors (69 companies). Bottom graph, industrial sectors reporting on different SDGs. In the first row (in red) we consider all ocean economic sectors together

In 2019, 60 percent of all the companies analysed committed to at least one SDG, but only seven percent (113 companies) reported on SDG14. The highest percentage of firms that included SDGs were found in Oceania, where 75% of companies analysed mention at least one goal. Companies prioritised 4 of the 17 goals in their annual sustainability reports (Fig. 3, top graph). Most companies were mainly focused on SDG 5-gender equality, SDG 13-climate action, and SDG 4-quality education. On the contrary, SDG 1-no poverty, SDG 2-zero hunger, SDG 10-reduced inequalities, and SDG 14-life below water were the least mentioned. Numbers changed when we consider just those companies working in the ocean economy. From the 69 companies included in this group, 20% (14 companies) reported on SDG14 just behind SDG 13-climate action and SDG 5-gender equality (Fig. 3, middle graph). The SDGs that received most attention in the reports can be considered as prioritised issues, or business areas where companies believe they can make a greater positive impact in contributing to the 2030 Agenda for Sustainable Development. Furthermore, these SDGs probably offer more standardised guidelines for reporting data and results, also following, in several cases, the evolution of legislative frameworks requiring the disclosure of non-financial information on specific issues (e.g. gender equality, and the main environmental issues).

Interest in SDG14 is growing. Despite being one of the least mentioned, the attention given to SDG 14 has more than tripled in two years (comparing 2018 data in 2019 reports with 2016 data in 2017 reports) and the number of companies referring to SDG14 increased from a mere 2.4% (2016) to 7.5% (2018). Moreover, when we compare this trend with trends for the other SDGs, it was evident that they follow the same growth pattern. Overall, our findings suggest that corporate commitment to sustainability is following a positive trend, the number of SDGs reporting grew and the consideration given to preserving marine and coastal ecosystems was increasing.

Different sectors have priorities in reporting on SDGs. The lower graph in Fig. 2 shows the percentage of companies reporting on SDGs vs the total sampled for each of the listed industries. When just the agricultural and the ocean economy sectors are considered, reporting on SDG14 rises above 20% (Fig. 3, bottom graph).

### <Response>: awareness, activation, and disclosing information

**Fig. 4:**
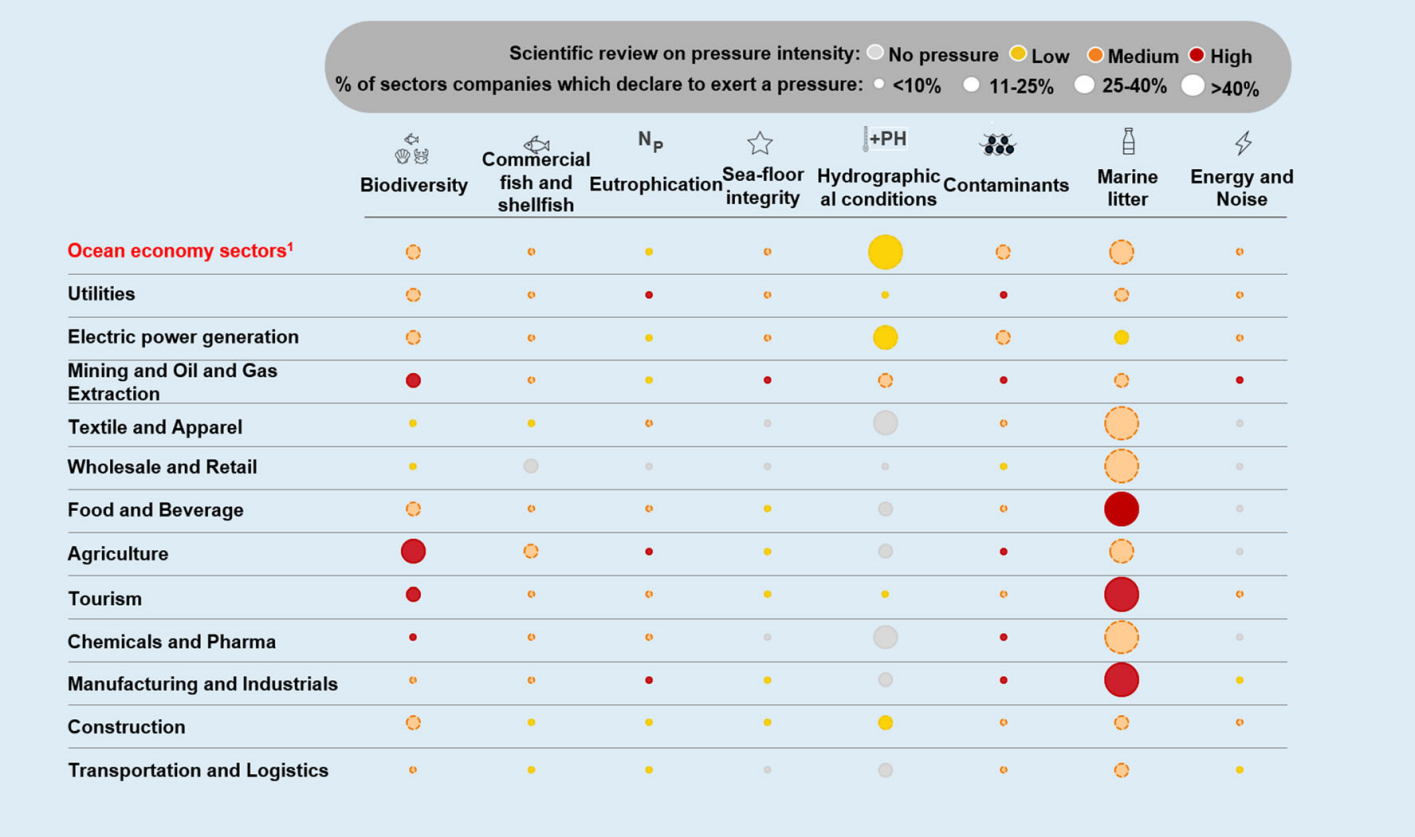
Pressure acknowledgment on a set of Good Environmental Status (GEnS) descriptors by different industrial sectors. In the first row (in red), we consider fisheries and aquaculture, maritime transportation, shipbuilding and repair, and ports and warehousing

51% of companies show awareness, albeit to varying degrees, when considering the potential pressures of their industries on the ocean (GEnS descriptors), but only a limited number acknowledge all the types of pressure (Fig. 4). Marine litter (mostly plastic), biodiversity, and hydrographical conditions (mostly associated with acidification) are the issues most frequently mentioned. Almost all sectors, to different degrees, are aware of the pressures directly or indirectly exerted on these descriptors. This level of concern can be considered as the result of growing attention from different stakeholders (e.g. media, policy makers, social movements, and consumers). Conversely, awareness of pressures on less publicised problems, such as over-exploitation of marine resources, eutrophication, seafloor integrity, and the introduction of energy in marine ecosystems is still limited, even though experts consider them to be relevant for most of the sectors. For example, hardly any of the companies from the mining and oil and gas extraction sectors mention seafloor integrity, and only a few food and beverage companies report on over-exploitation of marine resources. Finally, other GEnS descriptors (not appearing in Fig. 3) received no attention at all.

The ocean economy sectors follow approximately the same pattern; however, a higher percentage of firms reported on biodiversity, hydrographical conditions, and marine litter (while not properly identifying less publicised issues). For example, none of the companies belonging to ports and warehousing acknowledge its pressures on hydrographical conditions (e.g. changes in-depth, currents, waves, or turbidity of waters and coastal environment); similarly, fisheries and aquaculture companies seem unaware of the pressures exerted on seafloor integrity by their industry (e.g. caused by trawler fishing), and maritime transportation companies do not report on the introduction of energy in the ocean. Nonetheless, some companies in the maritime sectors mention less publicised topics, when relevant to their industries. This is the case for several fishing and aquaculture firms that appear to be aware of the problem of the over-exploitation of marine resources and for some maritime transportation companies that are concerned about contaminants.

Awareness issues showed mismatches. There are cases in which companies report pressures not considered significant for their industry by ocean scientists: 70% of maritime transportation firms report on hydrographical conditions. Similarly, one company in four in the retail and wholesale sector claims to be responsible for over-exploitation of marine resources, while scientists consider the pressures of these industrial sectors as a minor problem. These results show the existence of a mismatch between the review of pressures by ocean scientists and different industries on GEnS descriptors and the corresponding awareness of companies, as disclosed in their sustainability reports.

**Fig. 5:**
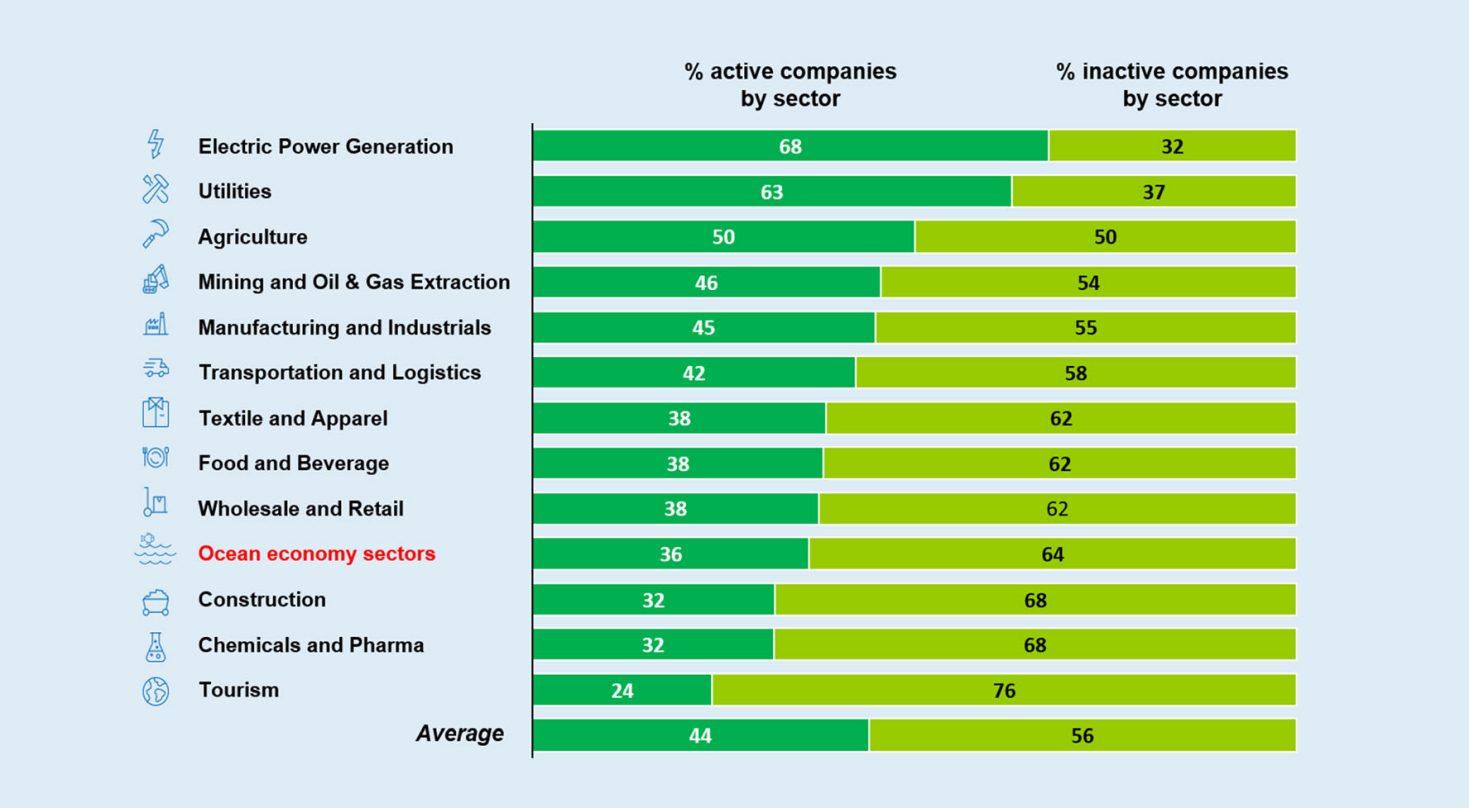
Percentage of companies by industrial sector that have actions that can, directly or indirectly, benefit ocean health. Ocean economy sectors (in red) are grouped in the figure

Awareness does not always imply response. Concerning activation, 44% of companies deploy activities that are beneficial for marine and coastal ecosystems. Companies can reduce or offset their negative pressures on marine and coastal ecosystems by deploying mitigating activities, whether they be product innovations, process innovations, or supply chain solutions. Electric power generation, utilities, and agriculture were the sectors with the highest percentages of active companies, contributing to the development of renewable energy sources and technological solutions for emission reduction (Fig. 5). When looking at the ocean economy sectors, about 36% of companies engage in activities that can benefit the ocean—the marine transportation sector showing the most observed initiatives.

Awareness and activation gates exist and need multilevel responses. Considering that 51% of companies are aware of some of their pressures on ocean health, and the fact that 44% of companies are active on ocean issues, shows that there are still cases where awareness does not correspond to activation. In other words, some companies mention ocean-related problems but do not report on activities carried out to mitigate them. What emerges is a gap between being aware of an environmental problem and responding through-specific initiatives. The evidence shows awareness and/or activation ‘gaps’ in most sectors.

- A cross-sectorial and common example of an awareness gap regards GHG emissions. About 5 companies in 10 carry out a carbon footprint assessment and cut their emissions, and more than 7 firms in 10 implement energy efficiency measures, but less than 1 company in 10 links emissions to ocean conditions. Therefore, some companies are already active in ocean preservation, but are unaware of the positive consequences of their activities on marine and coastal ecosystems.
- An example related to the activation gap concerns the chemical sector and microplastics. Even though marine litter is among the most acknowledged issues regarding ocean protection and one company in two in the chemical industry is aware of it, hardly any report on activities aimed at tackling microplastic dispersion in marine and coastal ecosystems, due to the lack of effective and commercially viable solutions.

**Fig. 6:**
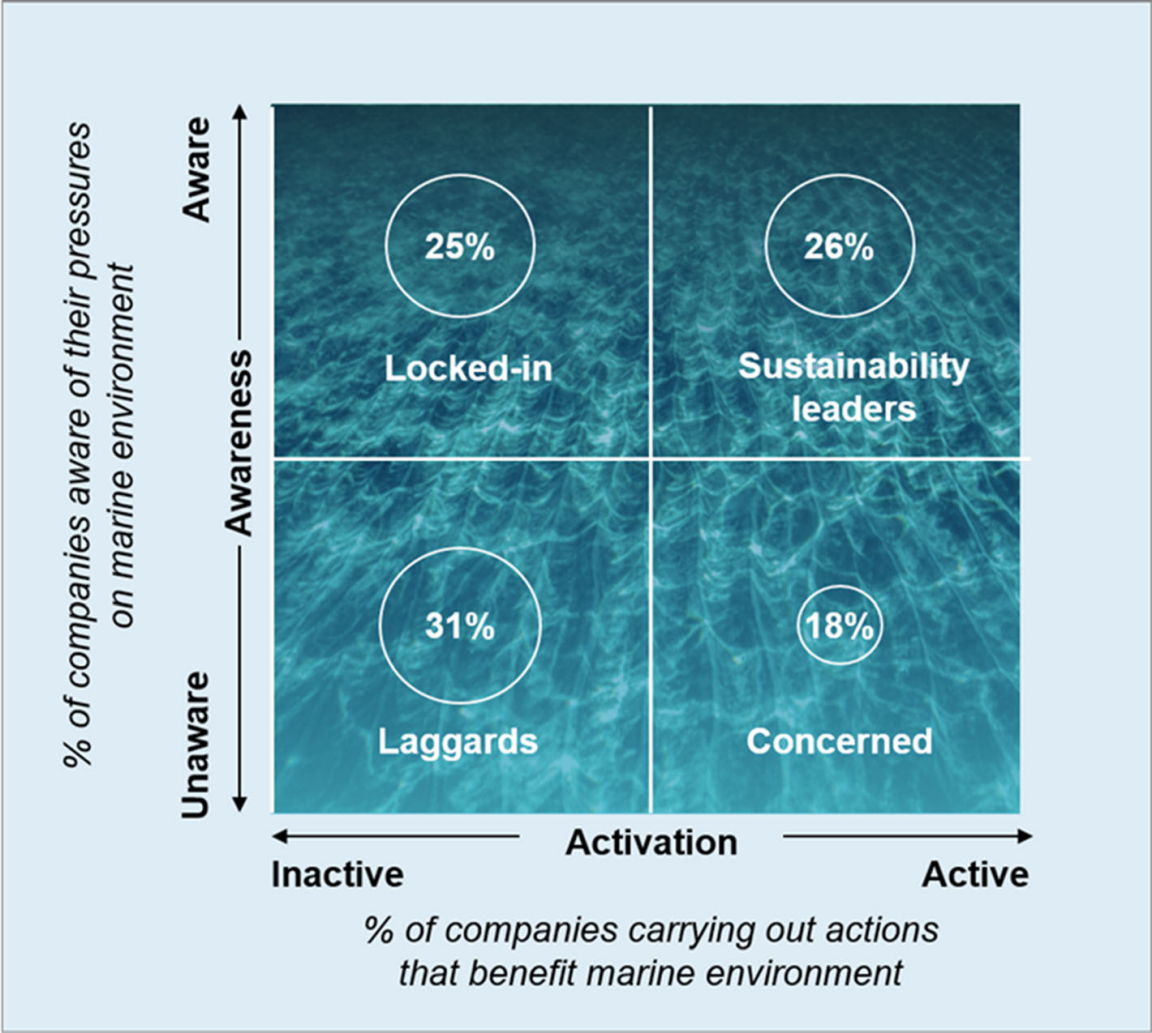
Awareness vs activation matrix

Building on these two dimensions, awareness and activation, companies can be categorised into four clusters: 26% of the companies in our sample are simultaneously aware and active (we call them sustainability leaders). These companies are active on the issues judged as medium or highly relevant by experts. On the other side, while 31% are unaware and inactive (laggards) they do not show any activation on any of the identified domains; 25% of companies are aware but inactive (locked-in), in the sense that they show awareness regarding the issues considered relevant by experts, but do not act to prevent or mitigate their pressure. The remaining 18% are unaware but active (concerned) (Fig. 6).

Transparency and disclosure practices are relatively widespread and provide information related to the sustainability strategies and initiatives developed by companies. From the 1664 companies that were analysed, 86% of these companies adopted at least one sustainability framework or standard. Nevertheless, none has specifically used an ocean transparency framework. Table 2, above shows the number of companies disclosing information in two groups that come from Fig. 6, the sustainability leader groups, and a second group that includes the other three. As additional evidence of their commitment to the sustainability agenda, companies refer to their inclusion in environmental, social, and governance (ESG) indices. Although not equally used by different regions and sectors, whenever included in ESG indices, companies mention this result in their sustainability reports as additional evidence of their commitment to the sustainability agenda (Table 2).

**Table 2:** Above: most popular sustainability transparency and disclosure standards, initiatives, and frameworks used by groups from the awareness/activation matrix (2019 percentages). Below: most popular indices found in non-financial reports (2019 percentages)

Standard	Sustainable leaders group	Rest of the groups
GRI	91	70
CDP	62	46
UNGC	52	33
IR	51	25
OECD	24	9
SASB	14	5
Global Reporting Initiative (GRI) designed to provide data and information to a wide range of stakeholders—from customers to the financial community CDP (former Carbon Disclosure Project) supporting companies in disclosing information on several aspects (carbon footprint, sustainable forest, water security management, and supply chains United Nations Global Compact (UNGC) a framework with principles on human rights, labour practices, the environment, and anti-corruption Integrated Reporting (IR) aimed at integrating economic, financial, and sustainability information related to corporate activities in one document OECD Guidelines (OECD) covering all key areas of business responsibility, including human rights, labour rights, the environment, bribery, consumer interests, as well as information disclosure, science and technology, competition, and taxation Sustainability Accounting Standards Board (SASB) designed to support businesses in providing investors with the most appropriate information on financial impacts of sustainability		

Standard	Sustainable leaders group	Rest of the groups
FTSE 4GOOD	31	21
DJSI	30	18
MSCI	28	14
ECPI	9	2

## Discussion

The oceans are essential for human existence and well-being; 3.3% of world gross domestic product came from this domain in 2017 and this makes oceans the world’s seventh-largest economy. Most of its contributions to people are not fully replaceable, and some are irreplaceable (IPBES 2019). However, as the trajectory of human expansion into the ocean accelerates, marine natural systems, and the goods and services they provide are rapidly deteriorating—following non-linearity models that make it difficult to reach sustainability targets such those incorporated in SDG14 (UN 2021). Significant changes in policies, organisations, and practices will be needed to reverse this tendency to environmental degradation, and this must include the response of corporations. Our findings illustrate early responses about how corporations are knowledgeable about their footprint (pressuring factors) in the marine environment, and they are starting to also recognise the strong dependence of the health of the ocean on their activities. Reversing this tendency will require transdisciplinary efforts to guide their activities, government incentives, and the involvement of civil society towards ocean stewardship (Lubchenco et al. 2016; Jouffray et al. 2020; Virdin et al. 2021).

Our first overview of the commitment by businesses to SDG 14 (compared to other SDGs) is shown as a snapshot of the level of attention among ocean economy companies to this specific SDG. The attention level is low and requires more dedication. Although we recommend the inclusion of SDG14 in corporate reporting as an indicator of this level of attention to ocean-related issues, we acknowledge that this measure is neither conclusive to evaluate the degree of awareness of companies concerning their pressures on marine ecosystems, or their dependence. Nor is it exhaustive when it comes to understanding business responses to addressing ocean challenges.

An in-depth analysis of the 2019 corporate sustainability reports highlighted that 51% of companies were aware to varying degrees of their pressures on ocean health, and 44% of companies were active on ocean issues—and this means that there are still cases where awareness does not correspond to activation. Two pieces of evidence resulted from this work. Firstly, a mismatch between the review by ocean scientists of industry pressures on GEnS descriptors and the corresponding consciousness of companies, as disclosed in their sustainability reports. Secondly, the gap between awareness by firms of their pressures on the ocean and related activities to reduce them. Unlocking both awareness and activation is crucial to engage companies in ocean sustainability. Nevertheless, unlocking activation is more complex than increasing awareness, and needs many resources and time. Unlocking activation requires organisational changes, a better internal ocean literacy, the availability of efficient and viable technological solutions, and the resolution of operational, economic, and organisational constraints that ‘lock-in’ companies.

In its recent paper, Virdin et al (2021) calls for higher voluntary corporate action on top of public policies, by engaging companies in collective action using multi-stakeholder platforms (green clubs), or its adherence to public guidelines and frameworks of best practices, claiming that all these actions should be grounded in science-based approaches (Österblom et al. 2020). By recognizing pressures on the ocean (“awareness”) and establishing voluntary action (”activation”) companies can help in the move from an ocean-based economy to this aspirational blue economy for the future. All industries and corporations analysed must go into this process by introducing recent new frameworks for corporate sustainability and reporting (Sardá et al. 2019).

To unlock awareness and activation, businesses must recognise that maintaining a healthy marine environment is a fundamental prerequisite for long-term operations, and that they have a shared responsibility to take the actions necessary to secure a healthy, resilient, and productive ocean. Understanding planetary boundaries (Rockström et al. 2009) and ocean planetary boundaries (Nash et al. 2017) appears as a basic need for every company that aims for long-term survival, and it will require strategic responses and instruments to improve internal ocean literacy (meaning an understanding of how the ocean affects companies, and how companies affect the ocean). Altogether, this is a call for a new framework of corporate sustainability, a new holistic approach that calls for the application of a ‘Business in Nature (BInN)’ view in line with the need for a new type of corporate sustainability for truly sustainable businesses (Dyllick and Muff 2016; Sardá and Pogutz 2019).

The BInN concept (Sardá and Pogutz 2019) calls for a profound social-technological transformation. To address global environmental challenges, businesses must engage and contribute to the development of a novel systemic transformation, a new techno-economic cycle, clean innovations, and responsible behaviour to create a new pathway concerning production-consumption mechanisms. Acknowledging the interdependencies of social-ecological systems (in our case, the ocean social-ecological system) is paramount. We believe that businesses must play a leading role in activating a virtuous cycle that links the goal of economic profitability with enhancing social capital (prosperity) and drastically reducing the pressures on ecological systems, both to maintain their functionality (effectiveness) and to ensure their resilience and the provision of ecosystem goods and services. In other words, we must recognise the idea that the global production-consumption machine is powering the world for development and welfare, and that nature and ecosystems also contribute and constitute its ultimate pillar. In this call, corporations should meet their business goals by creating a significant positive impact in societies without compromising the ability of natural systems to provide the resources and ecosystem services on which our well-being and that of other living species depend (Sardá and Pogutz 2019). A sustainable ocean economy (the newly desired and demanded blue economy) will only emerge when economic activity is in balance with the long-term capacity of ocean ecosystems to support this activity and remain resilient and healthy (The Economist 2015).

There is a lack of corporate reporting frameworks focused on ocean-related issues. However, sustainability frameworks are established and widely published. Companies committed to ocean sustainability have limited opportunities to report their strategies and achievements against ocean-specific targets and Key Performance Indicators (KPIs), which do not yet exist. None of these standards, initiatives, frameworks, or ESG indices are specifically designed to provide focused support or guidance on ocean-related transparency and disclosure. Therefore, companies willing to report on these issues are forced to prepare and adopt self-defined targets and indicators.

Reporting is unlikely to provide adequate evidence of how companies act. However, with the global demand for a Blue Economy, companies must start to understand how and why they perceive as material to them (and their stakeholders) regarding ocean issues and consequently worthy of inclusion in non-financial disclosure practices. Some preliminary research steps were initiated in this way to develop the Ocean Disclosure Initiative, a science-based framework aimed at increasing awareness of business pressures on marine and coastal ecosystems, gathering data to facilitate the assessment and disclosure of key performance indicators related to the ocean. In the future and regarding the aspirational Blue Economy, a disclosure tool aimed at helping companies along their sustainability path and providing stakeholders with additional insights in order to evaluate the ocean-related sustainability profile of companies and the associated risks, will be needed (OOF 2021).

Our findings suggest that greater awareness and activation can be boosted through new and dedicated initiatives aimed at promoting the disclosure of data regarding business pressures on marine ecosystems. Similarly, to the initiatives developed to tackle climate change, new instruments designed to support the reporting of pressures on the ocean and business mitigation initiatives would match growing needs for transparency and disclosure by companies, as well as the requests from stakeholders such as investors, consumers, and NGOs. Companies will need to develop a new ‘language’ that transforms the < pressure> into standardised and reliable metrics and KPIs that are functional for assessing the global risks related to ocean degradation, as well as measuring returns linked to the adoption of superior sustainability practices. The collection of ocean-based business data and information will help identify and select ocean leaders (those virtuous companies that have taken steps in managing and reporting their practices on marine ecosystems sustainability). The goal being the introduction of an Ocean Disclosure Index, which focuses on responding to the needs of investors to underpin a robust ESG analysis and facilitate the inclusion of ocean risks in investment decisions.

## Concluding remarks

This paper provides a comprehensive analysis of ocean governance in the private sector, a landmark description of the level of business awareness and response regarding the many direct and indirect pressures exerted on the oceans. To perform our research, we have used multiples methods and a thorough research path: reviewing secondary sources, and confronting data obtained from scientists and experts with data obtained from the exam of corporate sustainability reporting thanks to lexicometry and NLP.

The practice of sustainability reporting has become widespread among large multinationals, also thanks to the diffusion of standard guidelines (e.g. GRI and SASB) and mandatory frameworks (e.g. the EU Directive 2014/95). Despite some limitations that refers to the quality of information, the lacks transparency and standardization, sustainability reporting has become a common methodology to detect and analyse the level of awareness of companies and the types of ESG responses in management, accounting and sustainability fields.

Our study shows that although sustainability leaders exist (they are aware and have developed innovative solutions to mitigate their pressures on the ocean), most companies are locked-in, either because they are not aware of marine ecosystem problems, or because they are unable to respond with coherent and effective mitigation actions. This is a serious issue in a world moving towards a blue acceleration but delaying addressing those ocean challenges.

To address ocean sustainability, a common ambition plan with large collaborative agreements and significant changes in all industrial sectors will be needed. Business transformations both in the ocean economy sectors (extractive renewable, extractive non-renewable and, operational) and in the on-land sectors that indirectly are pressuring the oceans are necessary to reverse this situation. A sustainable ocean economy (the new desired Blue Economy) will only emerge when economic activity should be in balance with the long-term capacity of ocean ecosystems to support this activity and remain resilient and healthy. Companies must acknowledge their interdependence with the ocean by recognising that maintaining a healthy ocean is vital for long-term operations in all industries, not only in ocean-related industries.

A bridge between ocean sciences and business must be created; companies must be supported and accompanied in addressing and mitigating their most relevant direct and indirect pressures. The challenges are many and complex, but ocean sustainability can be mainstreamed and pursued with the immediate and effective mobilisation of the many interested parties. Reducing industrial pressures on the oceans and acknowledging the dependence of companies on its resources can be an important contributor to rebuilding marine life and restoring ocean ecosystems.

## Supplementary Information

Below is the link to the electronic supplementary material.

Supplementary file1 (PDF 829 kb)
