# Supplementary material for: Business for ocean sustainability: Early responses of ocean governance in the private sector
Source: Ambio. 2022 Oct 19;52(2):253–70. doi: 10.1007/s13280-022-01784-2 (PMC9755432; doi:10.1007/s13280-022-01784-2)

Ambio

Supplementary Information
This supplementary information has not been peer reviewed.

Title: Business for ocean sustainability: Early responses of ocean governance in the private sector

Supplementary Material 1. The DPSWR, a social-ecological accounting framework for the analysis of ocean sustainability.

2009 Nobel Prize winner, Elinor Ostrom, developed a comprehensive framework to analyse social-ecological systems (SES) and represent their dynamics. This framework extends the theory of common-pool resources and collective self-governance to areas of research that are still evolving. This approach relies heavily on systems ecology and the theory of complexity and incorporates other aspects from theories relating to the study of resilience, robustness, sustainability, and vulnerability. The SES approach also incorporates societal concerns such as equity, wellbeing, and environmental degradation.

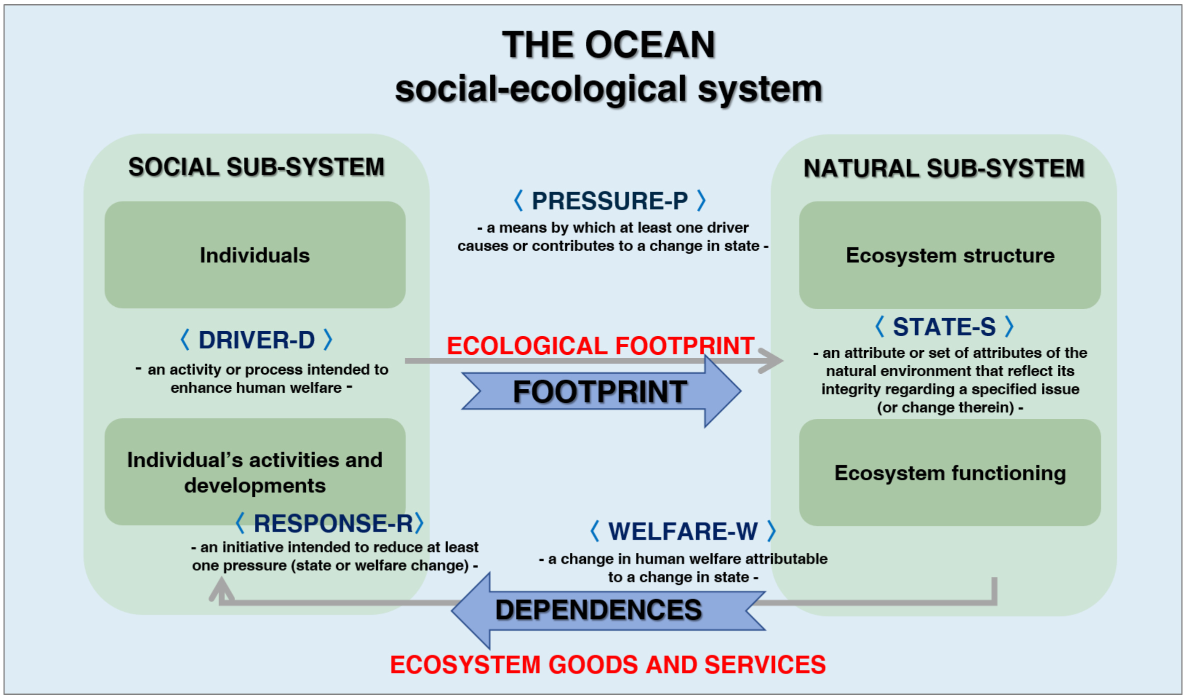

Diagram of the driver-pressure-state-welfare-response (DPSWR) framework used in this paper.

Cooper (2013) recently proposed an accounting framework to analyse the relations between social and ecological systems – the driver-pressure-state-welfare-response (DPSWR) framework (see enclosed figure). According to this approach, social sub-systems (humans, human capabilities, and their activities), are <drivers> of change (D). They put <pressure> (P) on the natural sub-systems (structural units and functions) that can alter their <state> (S) because of these pressures. This process, in turn, can translate into the degradation of fundamental natural resources used by humans (natural goods and ecosystem services), thus diminishing human <welfare> (W). The acknowledgement of such a process of degradation should induce humans to develop adequate <response> (R), for example, policies and innovative solutions that can address the ecological problems and help restore SES resilience. This proposal includes the components (structural and functional) of a social-ecological system, including the accountable

framework for the analysis. Other accounting frameworks can be followed, especially if do you want to be more precise in the adjustment of components (Elliott et al. 2017).

Supplementary Material 2. Definitions of industry sectors and good environmental status descriptors.

Supplementary Material 2a. Definition of industry sectors listed in Figure 3

Sector	Description
Shipbuilding and repair	Economic activities related to the manufacturing, repair, and maintenance of ships, boats, offshore platforms, and offshore supply vessels
Ports and warehousing	Economic activities related to the operation and management of ports, such as storage, loading and unloading activities, and cargo handling
Maritime transportation and logistics	Economic activities related to providing transportation of passengers and cargo, warehousing and storing goods, scenic and sightseeing transportation, and to supporting these activities
Fishing	Economic activities related to catching of live marine organisms for both food and other purposes
Aquaculture	Economic activities related to farmed production of living marine organisms for both food and other purposes
Utilities	Economic activities including energy transmission and distribution, water supply, sewage, and waste management
Electric power generation	Economic activities related to the use of fossil fuels (e.g., coal, oil, or gas) and renewable sources (e.g., hydroelectric, solar, wind) to produce electric energy
Mining and oil and gas extraction	Economic activities related to the extraction of naturally occurring mineral solids (e.g., coal and ore), liquid minerals (e.g., crude petroleum) and gases (e.g., natural gas); and beneficiating (e.g., crushing, screening, washing, and flotation) and other preparation at the mine site, or as part of mining activities
Textiles and apparel	Economic activities related to the production of textiles and fabrics from basic fibbers and to the transformation of these fabrics into clothing and other accessories
Wholesale and Retail	Economic activities that intermediate the sale of goods or services to retailers or to customers through multiple channels of distribution
Food and beverage	Economic activities related to food raw materials processing, packaging, and distribution. This includes fresh, prepared foods, as well as packaged foods, and alcoholic and non-alcoholic beverages. This sector includes tobacco product manufacturing
Agriculture	Economic activities related to soil cultivation, crop production, forest management, raising livestock (except living marine organisms), and in varying degrees, the preparation and marketing of the resulting products
Tourism	Economic activities related to hotels and motels, other traveller accommodations, food and drink service establishments
Chemical and pharmaceutical	Economic activities related to the basic preparations of chemicals, resins, synthetic rubber and fibres, pesticides, fertilisers, paints, coatings, adhesives, soaps, cleaning compounds and toiletries, and pharmaceutical and medicine manufacturing

Manufacturing and industrial	Economic activities related to traditional manufacturing (e.g., paper, wood, rubber, plastic, leather products) and to the production of industrial components and systems
Construction	Economic activities related to the construction of buildings, utility systems, highways, streets, bridges, and other heavy civil engineering
Water transportation	Economic activities related to water transportation of passengers and cargo using watercraft, such as ships, barges, and boats, and scenic and sightseeing water transport
Communications	Economic activities related to publishing and broadcasting (except internet), telecommunications, producing motion picture and sound recordings
Financials	Economic activities related to finance and insurance, meaning the creation, liquidation, or change in ownership of financial assets and/or facilitating financial transactions

Supplementary Material 2b. Definition and grouping of ocean economy sectors

Groups	Sectors	Description
Extracting renewable	Fisheries	Economic activity related to catch production
	Aquaculture	Farm production of living marine organisms for both food (e.g., seafood) and non-food purposes
Extracting non-Renewable	Offshore oil & gas	Exploration and extraction of crude petroleum and natural gas from offshore sources
	Seabed mining	Production and extraction of non-living resources in seabed or seawater from the continental shelf or the deep seabed (~4.000-6.000)
Operational	Coastal tourism	Tangible and direct facilities of maritime related tourism and leisure activities such as marine sports, aquariums, restaurants, hotels, accommodation, cruise shipping
	Water transport	Economic activity of transportation of freight and passengers (including linear services)
	Shipbuilding & repair	Manufacturing, repair, and maintenance of ships, boats, offshore platforms, and offshore supply vessels
	Ports & warehousing	Operation and management of ports, such as storage, loading and unloading activities and cargo handling
	Renewable Energy	Economic activities that use sea as an infrastructure: offshore wind energy (i.e., production of wind energy by generating electricity offshore) and ocean renewable energy (i.e., tidal energy and wave energy)
	Desalination	Economic activities including the process of seawater desalination for freshwater usage for agriculture irrigation, consumer, and commercial use.
	Genetic and medical resources	Economic activity related to the application of science and technology to living organisms from marine resources to produce goods and services (i.e., healthcare and pharmaceutical applications, agriculture, etc.)

Supplementary Material 2c. Definition of GEnS descriptors

GEnS Descriptors	Description
Biodiversity	The quality and occurrence of habitats and the distribution and abundance of species are in line with prevailing physiographic, geographic, and climatic conditions
Non-indigenous species	Non-indigenous species introduced by human activities are at levels that do not adversely alter the ecosystems
Commercial fish and shellfish	Populations of all commercially exploited fish and shellfish are within safe biological limits, exhibiting a population age and size distribution that is indicative of a healthy stock
Food Webs	All elements of the marine food webs, to the extent that they are known, occur at normal abundance and diversity and levels capable of ensuring the long-term abundance of the species and the retention of their full reproductive capacity
Eutrophication	Human-induced eutrophication is minimised, especially adverse effects thereof, such as losses in biodiversity, ecosystem degradation, harmful algae blooms, and oxygen deficiency in bottom waters
Seafloor integrity	Sea-floor integrity is at a level that ensures that the structure and functions of the ecosystems are safeguarded and benthic ecosystems are not adversely affected
Hydrographical conditions	Permanent alteration of hydrographical conditions does not adversely affect marine ecosystems
Contaminants	Contaminants are at a level that does not cause pollution effects
Contaminants in seafood	Contaminants in fish and other seafood for human consumption do not exceed levels established by EU legislation or other relevant standards
Marine litter	Properties and quantities of marine litter do not cause harm to the coastal and marine environment
Energy including underwater noise	Introduction of energy, including underwater noise, is at levels that do not adversely affect the marine environment

Supplementary Material 3. Direct and indirect pressures on GEnS.

GEnS Descriptors	Main sources of direct pressure	Main sources of indirect pressure
Biodiversity	Multiple and cumulative pressures on biodiversity derive from over-exploitation of natural species, introduction of non-indigenous species, eutrophication, seafloor destruction or alteration, changes in hydrographical conditions, pollution, climate change	Multiple and cumulative pressures on biodiversity derive from land-based sources of pollution, dumping grounds, and fluvial run-offs. Major land-based pressures to biodiversity are exerted by inorganic pollution, fertilisers, pesticides
Non-indigenous species	Natural invasion through waterways (also due to global warming and sea surface temperature increase), transportation by ships, intentional or unintentional introduction by aquaculture, including commercial species, bait, aquarium trade	Billions of pieces of marine litter (including plastics and microplastics) floating in the oceans are expected to be potential carriers for alien and invasive species
Commercial fish and shellfish	Over-exploitation, by-catch, direct and indirect impacts from fishing gears and trawler fishing, pollution, contaminants, and marine litter in seawaters are the main pressures on commercial fish and shellfish. Overfishing is the greatest single threat	
Food webs	Overfishing, eutrophication, modification of hydrographical conditions, introduction of non-indigenous species, pollutants and marine litter, and alteration of marine habitats can impact food webs and nutrient chains	Chemical and nutrients run-offs from rivers and land-based activities (e.g., agriculture), as well as pollution and contaminants from industrial activities can exert pressure on marine ecosystems (flora and fauna) and alter marine food webs and nutrient chains
Eutrophication	Excessive emission of nutrients through coastal wastewater treatment plants, discharges from aquaculture, ships and vessels, and tourism facilities. The largest emissions of organic matter in coastal areas originate from urban/domestic and industrial wastewaters entering marine environments through direct discharges	Organic and inorganic nutrient run-offs from rivers or from farming of animals, manure and fertilisers cause eutrophication of coastal areas
Seafloor integrity	Trawler fishing causes severe alterations of shallow (e.g., seagrass meadows) and deep-water ecosystems, reducing the number of species and the available habitats. Drilling, seabed exploitation, dredging, grounding, and anchoring exert additional significant threats to benthic and shallow water ecosystems	High-density marine litter accumulation on seafloor (continental shelves, canyons, and deep-sea-environments) is highly reported in the Mediterranean Sea, with plastic as the main marine litter component
Hydrographical conditions	Local and regional direct sources of pressure relate to sediment resuspension, and to altered conditions in localised hotspots (salinity, acidity, temperature)	GHG emissions from industrial, agriculture, and household activities influencing climate change and determining sea temperature rise, higher water acidity, decrease of oxygen

Contaminants	Hydrocarbons leaks and spills, biocides, and anti-fouling, coagulants, anti-foaming agents, and heavy metals are all present in ocean waters	Land-based sources of pollution such as wastewaters, discharge points and dumping grounds, fluvial run-offs, atmospheric deposition
Contaminants in seafood	Contaminants in sea waters, and especially heavy metals that cannot be degraded, represent a serious threat for marine species as well as for human consumption	Land-based sources of pollution, including contaminants and heavy metals, reach the sea through fluvial run-offs and atmospheric deposition and can contribute to seafood contamination. Recently discovered seafood contaminants relate to microplastics
Marine litter	Plastic, wood, metal, clothing, and paper waste originating from coastal household and municipal disposal, tourism facilities, pleasure craft and commercial vessels, are the main sources of marine litter and pollution. Plastic is by far the most common type of litter	Plastic, wood, metal, clothing, paper run-offs from rivers and land-based production and consumption activities
Energy including underwater noise	Energy, heat, noise, and vibrations introduced and/or discharged in water from exploration and exploitation activities, commercial transportation, and pleasure crafts exert an increasing pressure on the aquatic ecosystems	

Supplementary Material 4. <u>Panel of scholars and experts.</u>

The science-based analysis of the direct and indirect pressures by sector on ocean health was broadened with respect to previous work. A panel of 56 multi-disciplinary and international experts was involved. The panel of scholars and experts was selected according to a purposive sampling, with the aim of covering the different natural science and ocean domains. Experts had different natural science backgrounds and were identified from leading research institutes and universities across Europe, North and South America, and Australia.

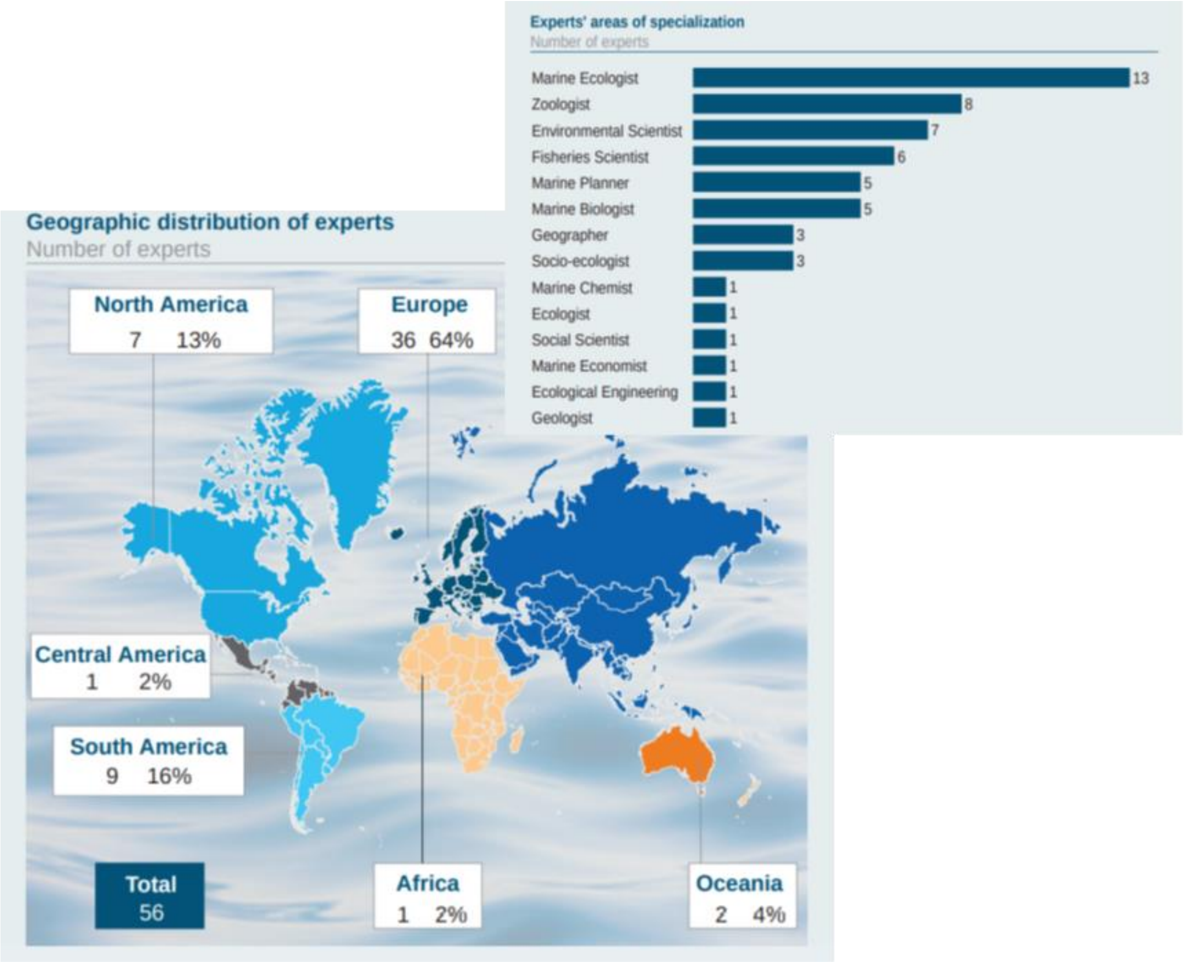

Panel of scholars by geographical area

Supplementary Material 4b. <u>Survey administered to the panel of experts.</u>

INTRODUCTION

The purpose of this survey is to collect ocean experts' opinions about the relevance of the pressures exerted by economic activities on ocean health, in particular on the Good Environmental Status (GES). The resulting science-based framework, will help us to assess companies' awareness of their sectors' pressures on marine and coastal ecosystems."

NEGATIVE PRESSURES RELEVANCE - SCORING

Please assess from 1 to 7 the RELEVANCE OF NEGATIVE DIRECT OR INDIRECT PRESSURES determined by the following sectors on EACH of the 11 GES descriptors where:

1 = No pressures exerted by the sector

2= Pressures are limited to local areas with low relative intensity, not suitable to determine significant alternations of ecosystems and biodiversity

3= Pressures are limited to local areas, presenting an intensity which may determine significant alterations of ecosystems and biodiversity

4= Pressures are spatially and/or temporally extended, presenting an intensity which may determine significant alterations of ecosystems and biodiversity

5= Pressures are spatially and/or temporally extended, with cumulative effects determining significant alterations of ecosystems and biodiversity

6= Pressures are spatially and/or temporally extended, with global and cumulative effects determining significant alterations of ecosystems and biodiversity

7= Pressures are spatially and/or temporally extended, with global and cumulative effects determining irreversible alterations of ecosystems and biodiversity

GLOSSARY

PRESSURE: a means by which an activity or process intended to enhance human welfare (driver) causes or contributes to a change in the attribute(s) of the natural environment (state)

DIRECT PRESSURES: pressures occurred through a direct interaction of an activity with an environmental component (e.g. Sea-floor integrity endangered by oil and gas drilling, sea-bed mining, fish trawling, etc.)

INDIRECT PRESSURES: pressures occurred through an indirect interaction of an activity with an environmental component (e.g. GHGs emissions determining an increase of sea temperature and acidification)

CUMULATIVE PRESSURES: pressures (positive or negative, direct and indirect, long-term and short-term) arising from a range of activities throughout an area or region, where each individual effect may not be significant if taken in isolation. Cumulative pressures may include a time dimension, since they should calculate the pressures on environmental resources resulting from changes brought about by past, present and reasonably foreseeable future actions.

Matrix for the input of responses from 1 (no pressure exerted by the sector) to 7 (Pressures spatially and/or temporally extended, with global and cumulative effects determining irreversible alterations of ecosystems and biodiversity)

	1: Biodiversity	2: Non-indigenous species	3: Commercial fish and shellfish	4: Food webs	5: Eutrophication	6: Sea-floor integrity	7: Hydrographical conditions	8: Contaminants	9: Contaminants in seafood	10: Marine litter	11: Energy incl underwater noise
Ship building and repair											
Ports and warehousing											
Maritime transportation and logistics											
Fishing											
Aquaculture											
Water transportation											
Utilities											
Electric power generation											
Mining and oil and gas extraction											
Textile and apparel											
Wholesale and retail											
Food and beverage											
Agriculture											
Tourism											
Chemicals and pharmaceuticals											
Manufacturing and industrials											
Construction											

Supplementary Material 5. Lexicometry analysis. Variables used and words accounted.

Variable 1. "Mention Ocean"

Keywords retrieved by NLP	Sample
"ocean" OR "sea" OR "ocean sustainability" OR "marine" OR "coastal"	Number of sustainability reports as of 2019<br>– 626 mentioning at least one of the KWs across industry sectors (excluding financials and technology and communication)<br>– 69 of blue economy sectors

Variable 2. "SDGs"

Keywords retrieved by NLP
**"SDG 14" OR "SDG14" OR "life below water" OR "sustainable development goal 14"**
"SDG 1" OR "SDG1" OR "sustainable development goal 1" OR "no poverty"
"SDG 2" OR "SDG2" OR "zero hunger" OR "sustainable development goal 2"
"SDG 3" OR "SDG3" OR "good health and well-being" OR "sustainable development goal 3"
"SDG 4" OR "SDG4" OR "sustainable development goal 4" OR "quality education"
"SDG 5" OR "SDG5" OR "sustainable development goal 5" OR "gender equality"
"SDG 6" OR "SDG6" OR "clean water and sanitation" OR "sustainable development goal 6"
"SDG 7" OR "SDG7" OR "affordable and clean energy" OR "sustainable development goal 7"
"SDG 8" OR "SDG8" OR "decent work and economic growth" OR "sustainable development goal 8"
"SDG 9" OR "SDG9" OR "industry innovation and infrastructure" OR "sustainable development goal 9"
"SDG 10" OR "SDG10" OR "sustainable development goal 10" OR "reduced inequalities"
"SDG 11" OR "SDG11" OR "sustainable cities and communities" OR "sustainable development goal 11"
"SDG 12" OR "SDG12" OR "responsible consumption and production" OR "sustainable development goal 12"
"SDG 13" OR "SDG13" OR "sustainable development goal 13" OR "climate action"
"SDG 15" OR "SDG15" OR "life on land" OR "sustainable development goal 15"
"SDG 16" OR "SDG16" OR "peace, justice and strong institutions" OR "sustainable development goal 16"
"SDG 17" OR "SDG17" OR "partnerships for the goals" OR "sustainable development goal 17"

Variable 3. "Pressure Awareness"

Topic	Keywords retrieved by NLP in proximity of 10 lexical items (words) one from the other: first word before ":" mandatory, then OR method is applied with proximity configuration
GES 1 – Biodiversity	"Biodiversity": "ocean" OR "sea" OR "marine" OR "coastal" OR "seawater"<br>"Ecosystem": "ocean" OR "sea" OR "marine" OR "coastal" OR "seawater"<br>"Fauna": "ocean" OR "sea" OR "marine" OR "coastal" OR "seawater"<br>"Flora": "ocean" OR "sea" OR "marine" OR "coastal" OR "seawater"
GES 2 – Non-indigenous species	"Indigenous species": "ocean" OR "sea" OR "marine" OR "coastal" OR "seawater"<br>"Alien species": "ocean" OR "sea" OR "marine" OR "coastal" OR "seawater"<br>"Native species": "ocean" OR "sea" OR "marine" OR "coastal" OR "seawater"

GES 3 – Commercial fish and shellfish	“Fish stock”: “ocean” OR “sea” OR “marine” OR “coastal” OR “seawater”<br>“Healthy stock”: “ocean” OR “sea” OR “marine” OR “coastal” OR “seawater”<br>“Overfishing”<br>“Sustainable fishing”
GES 4 – Food webs	“Food webs”: “ocean” OR “sea” OR “marine” OR “coastal” OR “seawater”<br>“Food chain”: “ocean” OR “sea” OR “marine” OR “coastal” OR “seawater”<br>“Food networks”: “ocean” OR “sea” OR “marine” OR “coastal” OR “seawater”
GES 5 – Eutrophication	“Eutrophication”:<br>“Phosphorous release”: “ocean” OR “sea” OR “marine” OR “coastal” OR “seawater”<br>“Nitrogen release”: “ocean” OR “sea” OR “marine” OR “coastal” OR “seawater”<br>“Algae bloom”: “ocean” OR “sea” OR “marine” OR “coastal” OR “seawater”
GES 6 – Seafloor integrity	“Floor integrity”: “ocean” OR “sea” OR “marine” OR “coastal” OR “seawater”<br>“Seabed”: “integrity” OR “preservation” OR “protection” OR “conservation” OR “restoration”<br>“Sea bottom”: “integrity” OR “preservation” OR “protection” OR “conservation” OR “restoration”<br>“Trawling”: “reduction” OR “avoidance”
GES 7 - Hydrographical conditions	“Hydrographical conditions”<br>“Emissions”: “ocean” OR “sea” OR “marine” OR “coastal” OR “seawater”<br>“Acidification”: “ocean” OR “sea” OR “marine” OR “coastal” OR “seawater”
GES 8 - Contaminants	“Contaminants”: “ocean” OR “sea” OR “marine” OR “coastal” OR “seawater”: “ocean” OR “sea” OR “marine” OR “coastal” OR “seawater”<br>“Toxic substances”: “ocean” OR “sea” OR “marine” OR “coastal” OR “seawater”<br>“Hazardous substances”: “ocean” OR “sea” OR “marine” OR “coastal” OR “seawater”<br>“Spills”: “ocean” OR “sea” OR “marine” OR “coastal” OR “seawater”<br>“Chemical”: “ocean” OR “sea” OR “marine” OR “coastal” OR “seawater”<br>“Metals”: “ocean” OR “sea” OR “marine” OR “coastal” OR “seawater”
GES 9 - Contaminants in seafood	“Contaminants”: “seafood”<br>“Toxic substances”: “seafood”<br>“Hazardous substances”: “seafood”<br>“Chemical”: “seafood”<br>“Metals”: “seafood”
GES 10 – Marine litter	“Marine litter”

	"Microplastics": "ocean" OR "sea" OR "marine" OR "coastal" OR "seawater"<br>"Microfibers": "ocean" OR "sea" OR "marine" OR "coastal" OR "seawater"<br>"Plastic": "ocean" OR "sea" OR "marine" OR "coastal" OR "seawater"<br>"Pollution": "ocean" OR "sea" OR "marine" OR "coastal" OR "seawater"
GES 11 - Energy including underwater noise	"Energy introduction": "ocean" OR "sea" OR "marine" OR "coastal" OR "seawater"<br>Noise"": "ocean" OR "sea" OR "marine" OR "coastal" OR "seawater"<br>"Vibrations": "ocean" OR "sea" OR "marine" OR "coastal" OR "seawater"<br>"Radiation": "ocean" OR "sea" OR "marine" OR "coastal" OR "seawater"

<u>Variable 4a. "Pressure action" keywords for all the sectors</u>

Topic	Keywords retrieved by NLP in proximity of 10 lexical items (words) one from the other: first word before ":" mandatory, then OR method is applied with proximity configuration
Organisational solutions	"Partnership" OR "initiative" OR "campaign" OR "alliance": "ocean" OR "sea" OR "marine" OR "coastal" OR "seawater"<br>"Stakeholder engagement": "ocean" OR "sea" OR "marine" OR "coastal" OR "seawater"<br>"Customer engagement": "ocean" OR "sea" OR "marine" OR "coastal" OR "seawater"<br>"Employee engagement": "ocean" OR "sea" OR "marine" OR "coastal" OR "seawater"<br>"Sustainability committee" OR "unit" OR "manager" OR "department"
Product innovation	"Eco design" OR "design": "environment"<br>"Product reuse""<br>"Biodegradable" OR "biomaterials" OR "bioplastic"<br>"Microplastics" OR "microfibers": "reduce" OR "reduction" OR "reducing" OR "avoid" OR "avoidance" OR "avoiding" OR "prevent" OR "prevention" OR "preventing"<br>"Plastic": ""reduce" OR "reduction" OR "reducing" OR "avoid" OR "avoidance" OR "avoiding" OR "prevent" OR "prevention" OR "preventing"<br>"LCA" OR "product life cycle"<br><u>Only blue</u>: "Anti-fouling" "Clean tech"<br><u>Only shipbuilding</u>: "Electric vehicle" "Biofuels"<br>"Transport": "sustainable" OR "sustainability"<br>"LNG": "sustainable" OR "sustainability"
Risk management	"Risk management": "ocean" OR "sea" OR "marine" OR "coastal" OR "seawater"<br>"Risk evaluation": "ocean" OR "sea" OR "marine" OR "coastal" OR "seawater"

	"Risk assessment": "ocean" OR "sea" OR "marine" OR "coastal" OR "seawater"
Process innovation	"CCS" OR "carbon capture"<br>"Power saving"<br>"Energy reduction"<br>"Energy efficiency"<br>"Recycling programs" OR "recycling initiatives" OR "recyclability" OR "upcycling"<br>"Waste disposal" OR "waste collection" OR "waste recovery" OR "waste reduction"<br>"Material efficiency"<br>"Wastewater management"<br>"Contaminants": "water"<br>"Cradle to cradle" "to gate"<br>"Afforestation" OR "Reforestation"<br>"Carbon credits" OR "Carbon offset"<br>"Emissions reduction"<br>"GHG reduction"<br>"Carbon footprint"<br>Only blue:<br>"Contaminants": "reduce" OR "reduction" OR "reducing" OR "prevent" OR "prevention" OR "preventing"<br>"Water footprint"<br>"PEF" OR "environmental footprint"<br>"Disclosure": ·ocean" OR "sea" OR "marine" OR "coastal and seawater"<br>"Eco label OR certification": "sustainable" OR "sustainability" OR "environment" OR "environmental sourcing"<br>"Procurement": "sustainable" OR "sustainability"<br>"Ballast water": "management" OR "treatment"<br>Only fisheries, ports and maritime transport:<br>"Electric vehicle"<br>"Biofuels"<br>"Transport": "sustainable" OR "sustainability"<br>"LNG": "sustainable" or "sustainability"<br>Only fisheries:<br>"Fishing": "sustainable" or "sustainability"<br>"Feed design": "sustainable" or "sustainability"<br>"MSC ASC"<br>"Wild fisheries": "monitor" OR "monitoring"<br>Only ports and maritime transport:<br>"Cold ironing" OR "Alternative marine power·<br>"Spills": "prevent" OR "prevention" OR "preventing" OR "recovery" OR "clean up" OR "cleaning up" OR "clean" OR "cleaning"

Variable 4b. "Pressure action" additional keywords for the so-called blue economy sectors

Topic	Keywords retrieved by NLP in proximity of 10 lexical items (words) one from the other: first word before ":" mandatory, then OR method is applied with proximity configuration

Regulation	“Regulation” OR “Policy”: “ocean” OR “sea” OR ”marine” OR “coastal” OR “seawater” OR “environment” OR “environmental” OR “sustainable” OR “sustainability”
Supply chain	“Audit”: “supplier/s” OR “distributor/s” OR “retailer/s” OR “provider/s”<br>“Certification”: “partner/s” OR “supplier/s” OR “distributor/s” OR “retailer/s” OR “provider/s” OR “supply chain·<br>“Code of conduct”: “supply chain” OR “supplier/s” OR “distributor/s” OR “retailer/s” OR “provider/s”<br>“Purchase contract”: “sustainability criteria” OR “sustainable criteria” OR “environment criteria” OR “environmental criteria”<br>“Traceability” OR “barcodes” OR “RFID tags”: “supply chain· OR “materials”<br>“Training”: “supplier/s” OR “distributor/s” OR “retailer/s” OR “provider/s”<br>“Disclosure”: “supply chain”<br>·Engagement” OR “collaboration” OR “cooperation”: “supplier/s” OR “distributor/s” OR “retailer/s” OR “provider/s”<br>“Sourcing”: “sustainable” OR “sustainability”<br>“Green procurement”

Variable 5. Reporting standards and sustainability indexes

Keywords
“Dow Jones Sustainability Index” OR “DJSI”<br>“ECPI”<br>“MSCI”<br>“FTSE4Good FTSE 4 Good”<br>“UN Global Compact”<br>“GRI” OR “Global Reporting Initiative”<br>“CDP” OR “Carbon Disclosure Project”<br>“IR” OR “Integrated Reporting”<br>“CDSB” OR “Climate Disclosure Standards Board Framework”<br>“SASB” OR “Sustainability Accounting Standards Board “

Supplementary Material 6. <u>Ocean economy data in terms of gross value added (GVA).</u>

In 2017, the Atlantic and Pacific oceans generated almost $1.8 trillion of gross value added (GVA); this represents 70% of the overall global ocean economy value added (Figure). In terms of employment, the Pacific Ocean has the largest share with 82.2 million employees (49% of the world ocean total), followed by the Indian (27.2%), and Atlantic (20.9%) oceans.

<u>Ocean economy (Gross Value Added-GVA) USD bn</u>

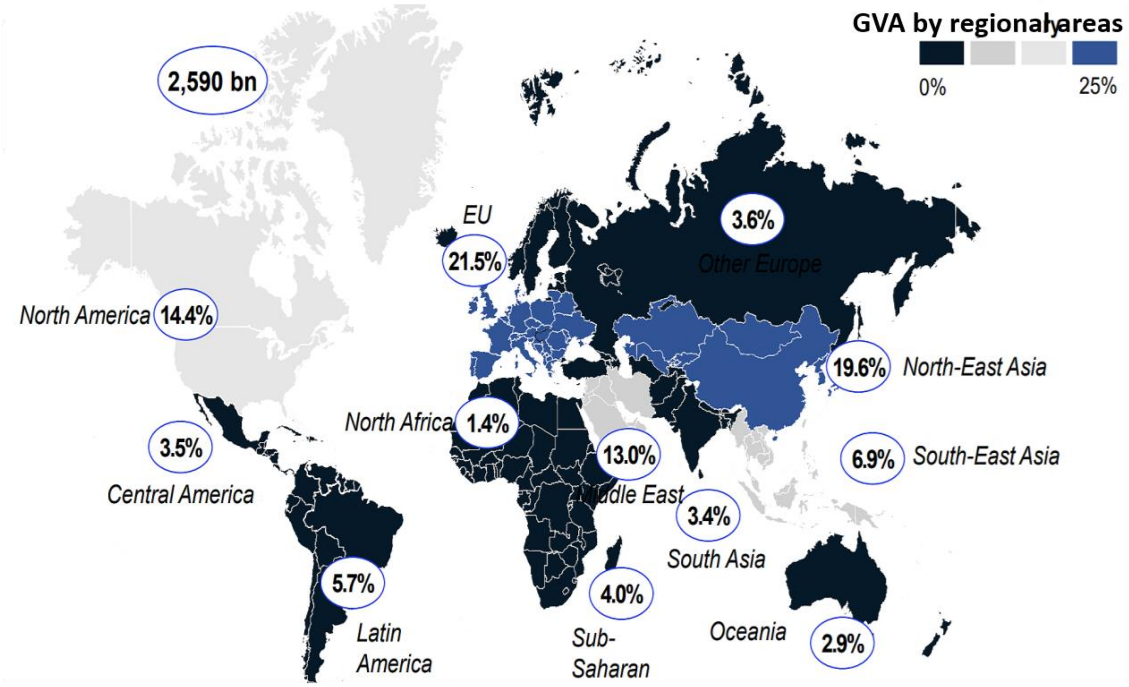

Supplement: Supplementary file 1 — Supplementary file1 (PDF 829 kb) [file 13280_2022_1784_MOESM1_ESM.pdf]
